# Urogenital Sinus Developmental Anomaly with Phallus and Accessory Phallic Urethra Presented as Disorder of Sex Differentiation in Female 

**Published:** 2014-01-01

**Authors:** Abhay S Bagul, Vijaya Sarathi, CM Bokade

**Affiliations:** Government Medical College and hospital, Nagpur, Maharashtra, India

A newborn presented on second day of life with enlarged phallus with urethral opening in it (Fig. 1), impalpable external gonads, normal vaginal opening, and normal anus (Fig. 2). Mother had no signs of virilisation and no history of androgenic drug intake in antenatal period. MRI showed normal uterus, bilateral ovaries and normal other abdominal organs. A phallic urethra was complete and 3F catheter can be introduced into the phallic meatus with difficulty, which entered the bladder about 0.5 cm. anterior to the perineal urethra (Fig. 3) as seen on cystoscopy. Genitogram showed normal vagina. Karyotyping was 46 XX. Blood glucose level, serum sodium, serum potassium, and 17 hydroxyprogesteron levels were normal. 

**Figure:**
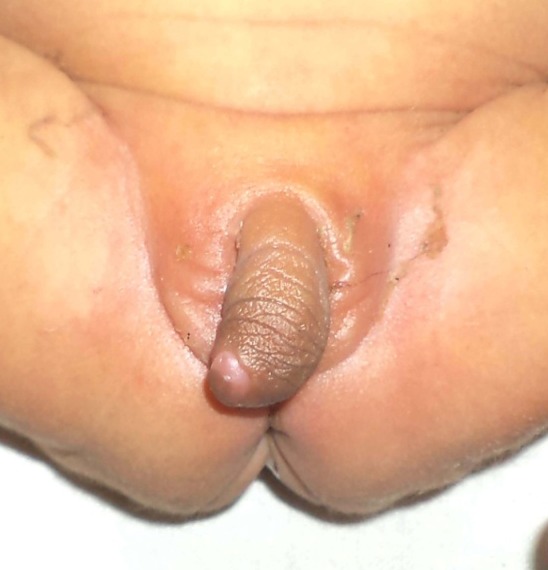
Figure 1: Enlarged phallus with accessory phallic urethra in female (46XX)

**Figure:**
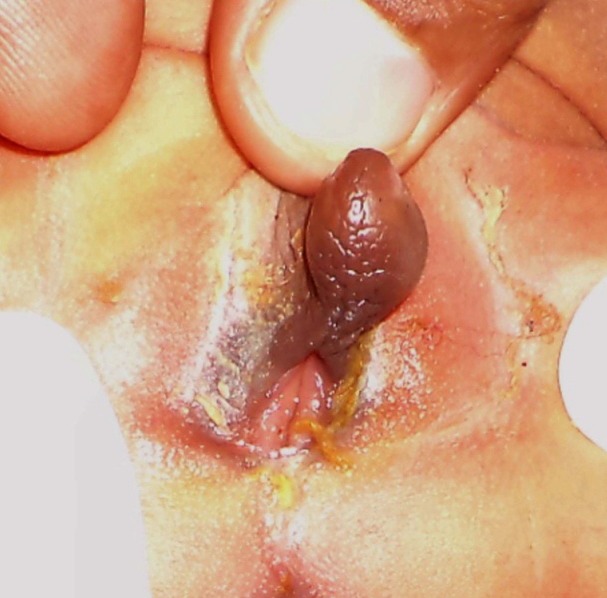
Figure 2: Absent labia minora and normal vaginal and anal openings

**Figure:**
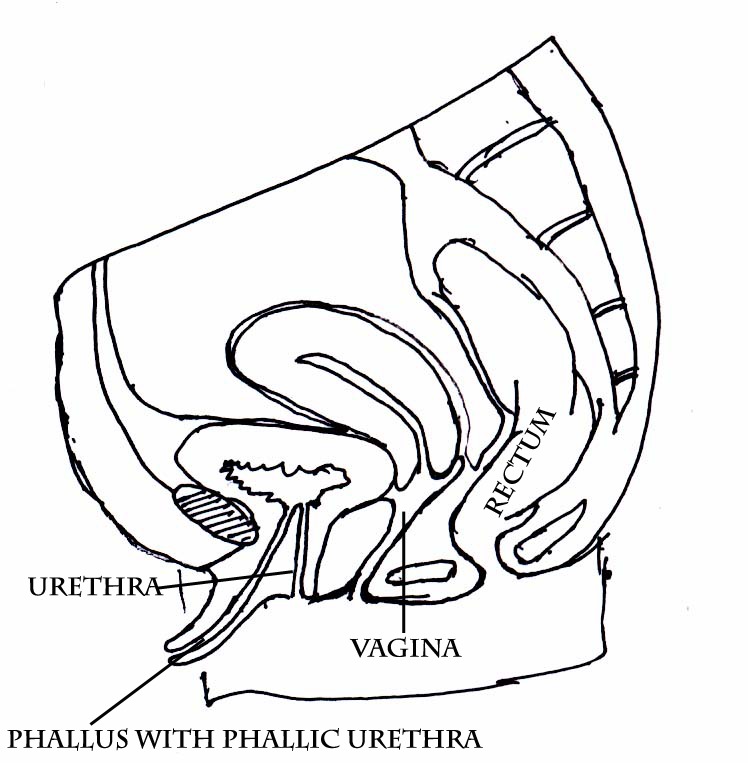
Figure 3: Schematic diagram showing phallus with accessory phallic urethra

A case of phallus with accessory phallic urethra in a genotypic female is uncommon and comes under group of 46XX DSD, previously called as over-virilisation of females. Causes of 46XX DSD are disorders of gonadal development, androgen excess and mullerian and local developmental defects. True fetal masculinisation is caused by too high levels of androgens either by excessive production as in fetal adrenal hyperplasia or by exposure to excessive maternal androgens. The degree of masculinisation of the external genitalia depends on time and severity of androgen exposure. In our patient, there was no androgen excess or exposure. In pseudo-masculinisation of the external genitalia, no relationship with virilising agents during pregnancy was established [1]. Though reported data showed frequent association of accessory phallic urethra with complex congenital malformations [2], persistent cloaca [3] and urinary obstruction [4], our patient didn’t have both any one of them. Abnormalities of mesodermal migration and differentiation in the caudal developmental field are thought to be responsible for such defects. Bellinger et al [5] reported 4 similar cases and suggested an abnormal descent of the mullerian ducts results in posterior displacement of the vaginal introitus; allows the accessory phallic urethra to form; and results in an unusually prominent phallus.

## Footnotes

**Source of Support:** Nil

**Conflict of Interest:** None

